# Comparative Analysis of Machine Learning Models for Image Detection of Colonic Polyps vs. Resected Polyps

**DOI:** 10.3390/jimaging9100215

**Published:** 2023-10-09

**Authors:** Adriel Abraham, Rejath Jose, Jawad Ahmad, Jai Joshi, Thomas Jacob, Aziz-ur-rahman Khalid, Hassam Ali, Pratik Patel, Jaspreet Singh, Milan Toma

**Affiliations:** 1 New York Institute of Technology, College of Osteopathic Medicine, Old Westbury, NY 11568, USA; aabrah21@nyit.edu (A.A.); rjose02@nyit.edu (R.J.); jahmad@nyit.edu (J.A.); jjoshi06@nyit.edu (J.J.); tjacob05@nyit.edu (T.J.); akhali16@nyit.edu (A.-u.-r.K.); 2 Division of Gastroenterology, Hepatology, and Nutrition, Department of Internal Medicine, Brody School of Medicine, East Carolina University, Greenville, NC 27858, USA; alih20@ecu.edu; 3 Department of Gastroenterology, Northwell Mather Hospital, Port Jefferson, NY 11777, USAjsingh19@northwell.edu (J.S.)

**Keywords:** machine learning, image detection, polyps, colonic, resected, cancer, image classification

## Abstract

(1) Background: Colon polyps are common protrusions in the colon’s lumen, with potential risks of developing colorectal cancer. Early detection and intervention of these polyps are vital for reducing colorectal cancer incidence and mortality rates. This research aims to evaluate and compare the performance of three machine learning image classification models’ performance in detecting and classifying colon polyps. (2) Methods: The performance of three machine learning image classification models, Google Teachable Machine (GTM), Roboflow3 (RF3), and You Only Look Once version 8 (YOLOv8n), in the detection and classification of colon polyps was evaluated using the testing split for each model. The external validity of the test was analyzed using 90 images that were not used to test, train, or validate the model. The study used a dataset of colonoscopy images of normal colon, polyps, and resected polyps. The study assessed the models’ ability to correctly classify the images into their respective classes using precision, recall, and F1 score generated from confusion matrix analysis and performance graphs. (3) Results: All three models successfully distinguished between normal colon, polyps, and resected polyps in colonoscopy images. GTM achieved the highest accuracies: 0.99, with consistent precision, recall, and F1 scores of 1.00 for the ‘normal’ class, 0.97–1.00 for ‘polyps’, and 0.97–1.00 for ‘resected polyps’. While GTM exclusively classified images into these three categories, both YOLOv8n and RF3 were able to detect and specify the location of normal colonic tissue, polyps, and resected polyps, with YOLOv8n and RF3 achieving overall accuracies of 0.84 and 0.87, respectively. (4) Conclusions: Machine learning, particularly models like GTM, shows promising results in ensuring comprehensive detection of polyps during colonoscopies.

## 1. Introduction

Colon polyps are mucosal protrusions within the colonic lumen. Based on their size, they are categorized as minuscule (5 mm or less), small (6–9 mm), and large (1 cm or greater in diameter) [1]. Despite being predominantly asymptomatic, these polyps are commonly identified during screening colonoscopies. Predominantly originating from the mucosa, colon polyps can be benign, adenomatous, or serrated [2]. The prevalence of adenomas among North American patients aged 50–75 was 30.2% [3]. Colorectal cancer is the third most common malignant disease around the world, with 1.85 million new cases per year [4]. While a malignant potential is inherent to certain polyps, it is crucial to note that not all polyps undergo malignant transformation. Adenomatous and serrated polyps present malignant potential, whereas hyperplastic, post-inflammatory, and hamartomatous polyps generally do not [5]. Given the potential for malignancy in certain polyps, standard procedure dictates their resection, followed by a detailed pathological analysis to ascertain their histological profile. This protocol is often underscored because over 95% of colorectal cancers are derived from adenomatous polyps [1]. Most notably, colonic adenomas serve as the precursor to a significant proportion of colon cancers, exhibiting progression in size and degrees of dysplasia [6]. Timely identification and intervention concerning polyps are pivotal as they directly impact colon cancer mortality rates because colon cancer is the second most common cause of cancer death in the United States [7].

According to the current guidelines, people at an average risk of colon cancer in the general population are advised to start colon screening at the age of 45 [8]. There are also screening guidelines for people who have relatives affected by colon cancer. These recommendations suggest that individuals should start their screenings at the age of 40 or ten years before a first-degree relative was diagnosed with colon cancer, whichever comes first. Additionally, if there are two first-degree relatives who had been diagnosed with colon cancer at any age, it is advised to begin screenings without delay [9]. Colon cancer screening is usually individualized after the age of 75, considering the patient’s screening history and health [10].

Colonoscopy is a standard diagnostic and therapeutic tool to detect any colon polyps that could potentially be malignant. However, a meta-analysis of 43 publications with more than 15,000 tandem colonoscopies shows a miss rate of 26% for adenomas, 9% for advanced adenomas, and 27% for serrated polyps [11]. Based on previous literature, factors that may contribute to missed polyps include inadequate bowel preparation, suboptimal procedure techniques, and incomplete removal of polyps [12,13]. Methods to ensure thorough polyp removal and reduce the incidence of interval colon cancer include cecal intubation time, satisfactory bowel preparation, and a colonoscopic withdrawal period lasting at least 6 min or more [12]. However, colon polyps might still go undetected during routine surveillance.

### Related Works

Machine learning image detection has made significant strides in the medical field, especially in colonic polyp detection. Several studies have explored the application of convolutional neural networks (CNNs) and deep learning techniques to detect polyps in colonoscopy images automatically, illustrating their clinical importance in minimizing undetected polyps and enhancing polyp detection rates [14,15,16]. Research conducted at Xiangya Hospital of Central South University analyzed 681 colonoscopy images. Utilizing the DeFrame system, they achieved an impressive 100% recall and 80% sensitivity. This system effectively identifies polyps with diverse morphologies and locations within colonoscopy footage [13]. In addition, Wan et al. proposed an attentive YOLOv5 model, which achieved a precision of 0.915 and recall rate of 0.899 on the Kvasir dataset [16]. Misawa et al. developed a 3-D convolutional network model, which worked nearly in real time and achieved a sensitivity of 90%, specificity of 63.3%, and accuracy of 76.5% with a training set of 411 short videos [17]. These artificial intelligence (AI)-based systems have shown promising results in improving the detection rate of polyps, which is critical for early diagnosis and intervention in colorectal cancer.

A study completed in 2020 utilized YOLOv2 to create an algorithm that can be used to identify polyps [18]. In this study, there were four datasets. Each dataset allowed for a unique sample of colonic polyps that can be detected. The various samples included images of polyps or videos of colonoscopies that displayed polyps. The efficacy of the algorithm was later tested via a trial to detect colonic polyps in 15 unaltered colonoscopy videos. The results in this study showed promising efficacy for using YOLOv2 for detecting colonic polyps. More specifically, YOLOv2 was able to detect seven more polyps in addition to the thirty-eight that were already detected by endoscopists [18]. The dataset of unaltered colonoscopy times was used to calculate the per-image sensitivity and the false positive rate of the created YOLOv2 algorithm in detecting colonic polyps. The per-image sensitivity was 88.3% and the false positive rate was 6.2% [18].

Another study completed in 2022 utilized a CNN deep learning model to assess colorectal polyps using a Single Shot MultiBox Detector on colonoscopy images [19]. It initially employed a guided image filter and a dynamic histogram equalization technique that filtered and enhanced the colonoscopy images. The study used a total of 27,508 images from a website called “Virtual Pathology at the University of Leeds” and divided them into a testing and training set [19]. To train the CNN model, they used 15,418 images, among which 3254 were proven to be polyps and 4001 of normal colonic mucosa [19]. To train the data, they used 7077 images, among which 1172 images contained polyps. The trained network classified adenomas with a 96% sensitivity and a 86% accuracy in white light images. The proposed model used in the study achieved an accuracy of 92% [19].

Research on machine learning polyp detection models is promising; however, further research and validation on larger and more diverse datasets are essential to establish the generalizability and robustness of these AI-driven solutions [20]. For example, the selection of the most suitable model can be a major challenge. In the current study, this issue will be addressed by comparing three prominent machine learning image identification models: GTM, RF3, and YOLOv8n. One of the study’s ultimate objectives, when evaluating the effectiveness of each model, is to determine its performance in its default configuration, i.e., how well it operates without any modifications made by the end user. The focus will be on the ability of each model to become trained and tested on the same set of images to accurately differentiate between normal colonic tissue, resected polyps, and polyps. Even though all three models are being compared, not all of them provide the same information as GTM is a pure classification model where it classifies an image into a category, whereas RF3 and YOLOv8n are detection models that detect different items/objects in an image and can quantify how many of the items/objects are in the image. The current study is of paramount importance given that colon cancer ranks as the second leading cause of cancer-related deaths in the US and the global new colorectal cancer cases are predicted to reach 3.2 million by the year 2040, while colonic polyps can be missed easily due to a multitude of reasons [21].

## 2. Methods

The methods section of this study includes several key subsections that outline the various steps and processes involved in conducting the research. These sections are as follows: (1) Data Collection and Preprocessing: This subsection details how data were collected for the study, including any relevant sources or datasets used. It also describes any preprocessing techniques applied to ensure data quality and consistency. (2) Google Teachable Machine Setup and Image Classification: Here, we explain the setup process for using Google Teachable Machine to train our image classification models. We describe any specific parameters or settings utilized during training. (3) RoboFlow 3.0 Object Detection Setup and Image Classification: In this subsection, we provide an overview of how RoboFlow 3.0 was configured for object detection tasks in our study, along with explanations of the specific approaches employed. (4) You Only Look Once version 8 Object Detection Setup and Image Classification: This section outlines the procedures used to set up You Only Look Once version 8 for object detection purposes in our research project. (5) Metrics from Different Models and Their Meanings: Here, we present a thorough analysis of metrics obtained from all models used throughout this investigation. The meaning behind these metrics is explained within context to help interpret their significance effectively. (6) Assessing External Validity of All Models: This subsection focuses on evaluating the external validity of the models employed in this study.

### 2.1. Data Collection and Preprocessing

**Figure 1:**
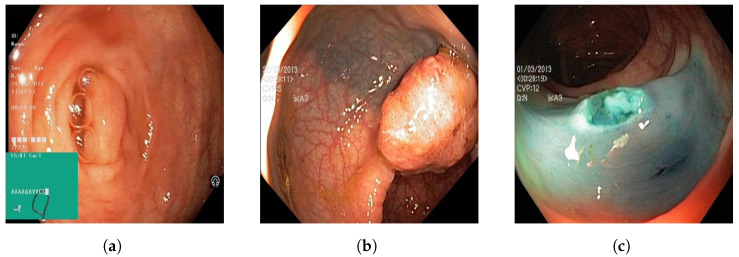
Sample images of (**a**) normal colonic tissue, (**b**) pylop, and (**c**) resected pylop.

An open-source online gastrointestinal endoscopy database was used to train the machine learning models. The database used in this research is titled “HyperKvasir”, curated by Borgli et al. and sourced from Simula Datasets [22]. The dataset consists of 110,079 images of the GI tract, out of which 10,662 are labeled and 99,417 are unlabeled images. The labeled image dataset consisted of anatomic landmarks such as the cecum, ileum, and retroflex-rectum. The dataset also has pathologic findings, such as polyps, as well as therapeutic interventions, such as dyed-resection margins. Sample images from all three classes are outlined in Figure 1. The complete data set, containing all the images utilized for the three machine learning models, can be accessed via [23].

A total of 601 images were used to train the image classification models to train the models on differentiating between normal colonic tissue, polyp, and resected polyp. Further, 201 images of the cecum were used as “normal colonic tissue”. The model was trained using a dataset consisting of 200 images of polyps found in various sections of the lower gastrointestinal tract, along with an additional set of 200 images depicting dyed-resected polyp margins. The purpose of this training was to differentiate between polyps that were still intact and those that had been surgically removed. All the images were cropped to ensure a 1:1 aspect ratio (~640 × 640 pixels). All the images were also ensured to be the same format of Joint Photographic Experts Group (JPEG). Images that at least two authors deemed to be subjectively obstructed or suffering from over/underexposure were subsequently excluded from the HyperKvasir dataset, resulting in the final selection of 601 images. Three different image classification models were trained: GTM, RF3, and YOLOv8n.

In order to assess external validity of the models, 90 extra images (30 normal, 30 polyp, and 30 resected polyp) were extracted from the HyperKvasir database. All three models do not have access to these 90 extra images, and the 90 images are externally fed into all three models. All of the images had the same pre-processing steps as the images that were used to train each model. After each image is fed into each model, new confusion matrices are generated and precision, recall, F1 score, and accuracy are calculated as described previously. Once each of the 90 images is fed into each of the three models, each model outputs a confidence value indicating how confident the model is in making this prediction. The mean and standard deviation for each class for each model are calculated.

### 2.2. Google Teachable Machine Setup and Image Classification

Once the dataset was processed, Google Teachable Machine’s web tool was used to develop an image classification model for polyp detection. GTM is an online platform that allows users to train, test, and deploy machine learning image classification models quickly [24]. To utilize the Image classification model in GTM, all 601 images from the pre-processed dataset were imported into GTM. The images were labeled into 3 separate classes: normal, polyp, and resected polyp according to their labels assigned in the dataset. The data were trained for 300 epochs with a batch size of 32 and learning rate of 0.0001. Epoch refers to the number of times each image is fed through the training model. Batch size refers to the number of images used in one iteration of training. The learning rate determines how much the model’s parameters are adjusted in each update step during training. The batch size of 16 and learning rate of 0.001 are the default settings from GTM; however, fine adjustment of the program has shown that a batch size of 32 had a higher accuracy without significant differences in training time. In addition, the learning rate is small (0.0001) in order to ensure that the model gradually adjusts its parameters; prior testing with the dataset has shown better accuracy with lowering of the learning rate from 0.001 to 0.0001. GTM also does not provide further documentation on the specific deep learning architecture for the image classification model. Since there are 601 images altogether and 32 images per batch, there are a total of 19 batches. Once all batches go through the dataset, one epoch is complete. GTM also generates its own evaluation train/test split as 85% of the images are split into training samples (170 images per class) and 15% into test samples (30 images per class). This split cannot be changed by the user.

### 2.3. RoboFlow 3.0 Object Detection Setup and Image Classification

In order to generate an image classification model using RF3, the images first need to be annotated with the correct labels (i.e., normal, polyp, or resected polyp). Computer Vision Annotation Tool (CVAT) was used to annotate the images. CVAT was used to annotate normal colonic tissue, polyps, and resected polyps for all 601 images. The annotation data (class and position) of the image were exported as text files from CVAT. The text files were uploaded along with the 601 images onto the RoboFlow website. RoboFlow also generates its own train/validate/test split; however, the end user can change the split. For the current study, the train/validate/test split was not changed from the default setting, with 70% of the images for training (421 images in total), 20% of the images for validation (120 images in total), and 10% for testing (60 images in total, split as 24 images for “normal”, 19 images for “polyp”, and 17 images for “resected polyp”). RoboFlow also allows the user to preprocess the data on their own website; however, since the images were already pre-processed, this was not completed on RoboFlow. Finally, the images were trained using “RoboFlow 3.0 Object Detection (FAST)”. RoboFlow also recommends using a pre-trained benchmark model to give the model prior information to improve performance; Microsoft Common Objects in Context version 7 (MS COCO v7) was used as the pre-trained benchmark. RoboFlow automatically determines the number of epochs necessary for proper training of the model; the current model was trained for 300 epochs.

### 2.4. You Only Look Once Version 8 Object Detection Setup and Image Classification

Prior to using YOLOv8n, the images needed to be annotated correctly as with the RF3 model. The same CVAT annotations were used as above for all 601 images. The annotation data of the images were exported as text files from CVAT, and the text files along with the images were uploaded onto the YOLOv8n website. The same 70–20–10 train–validate–test split used for RF3 was exported with the YOLOv8n format. This downloaded file contains images and labels that are split into training, validation, and testing groups. YOLOv8n is implemented as a deep learning model in Python, and the model architecture and weights must be generated or downloaded using Python code. Google Colaboratory (Colab) was used to write and execute the code to generate a YOLOv8n model. Before creating and training the YOLOv8n model, it is imperative to import all the images and labels into a specific Google Drive folder. In the current study, there were two overarching folders named “images” and “labels”, and, within each folder, there was a folder for “train”, “val”, and “test”, which contained the images and labels for each split (i.e., images for the training split go into the “images” → “train” folder). Once the folders are configured correctly, a .YAML file will be used to ensure that the correct images train, validate, and test the model. Before using the .YAML file, make sure to open the file and rename the path to the proper folder name. After configuring the .YAML file, the YOLOv8n model can be created and trained. The YOLOv8n model used in this study is based on the Ultralytics YOLOv8n.0.137 implementation; specifically, the YOLOv8n nano (YOLOv8nn) model was used for the current study. The model was set to train for 300 epochs with early stopping patience of 50 epochs, and a batch size of 32 is used. “Early stopping patience of 50 epochs” means that, during training, the model will monitor for changes in a specific metric (mAP), and, if there is no improvement in this metric for 50 or more epochs, the training process will be stopped early. All other settings were left as is. The AdamW optimizer is used for training with a learning rate of 0.001429, momentum of 0.9, and weight decay of 0.0005. The optimizer (AdamW) is responsible for updating the model’s parameters (weights and biases) during training to minimize the loss function and improve the model’s performance with each iteration. Learning rate is a hyperparameter that refers to the step size for adjusting model parameters during training, so smaller learning rate means smaller steps in adjusting the model parameters, which can extend training times. Momentum is a hyperparameter that adds inertia to the parameter updates, allowing the optimizer to gain speed in the right direction, thus reducing oscillations during training. Finally, weight decay is technique that reduces the likelihood that the model just memorizes features of the images during training. The YOLOv8n model consists of 225 layers and has a total of 3,011,433 parameters. The training data contain 421 images; the validation data consist of 120 images. Data augmentation techniques, including blur, median blur, grayscale conversion, and Contrast Limited Adaptive Histogram Equalization (CLAHE), are applied to the training data using the Albumentations library. The rest of the 60 images will be used for testing and generating metrics. Table 1 shows the summary for the main settings applicable for GTM, YOLOv8n, and RF3. RF3 does not allow for changes in batch size and learning rate and the user does not gain access to the actual batch size and learning rate.

**Figure 2:**
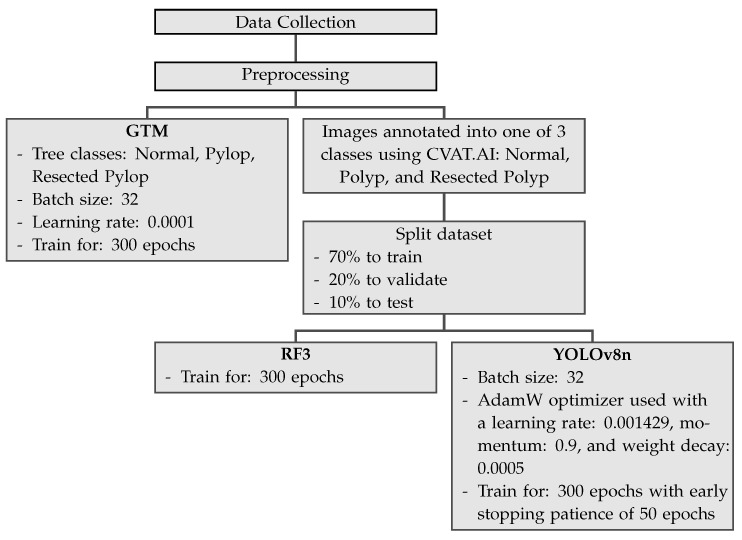
Pipline demonstrating the workflow for each machine learning model.

Figure 2 shows the pipeline for the three different machine learning models used in the current study.

### 2.5. Metrics from the Different Models and Their Meanings

**Figure 3:**
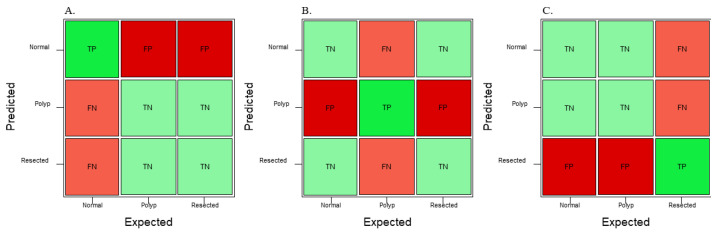
Sample confusion matrix showing TP, TN, FP, and FN if predictions are required for (**A**) normal colonic tissue, (**B**) polyp, and (**C**) resected polyp.

GTM generates its own confusion matrix, accuracy data, as well as train–test performance graphs; however, it does not provide extra parameters, such as precision, recall, and F1 score. The confusion matrices generated by GTM are not comparable to the graphs generated by RF3 and YOLOv8n because the confusion matrix in GTM is made of a random 15% of the dataset, so the same 10% (60 images) generated by RF3 train–validate–test split was used to generate another confusion matrix as well as metrics such as precision, recall, and F1 score. RF3 does not generate its own confusion matrix; the images from the test split were fed back into the RF3 model and the True Positive (TP), True Negative (TN), False Positive (FP), and False Negative (FN) were each recorded similar to Figure 3. The generated confusion matrix was then used to generate different metrics, such as precision, recall, F1 score, and overall accuracy. YOLOv8n generates its own confusion matrix as well as precision and loss graphs; it also provides information on precision, recall, and mAP. In order to ensure that metrics from all three models are comparable to each other, precision, recall, and F1 score are calculated from a confusion matrix. Precision, recall, and F1 score are used to analyze the performance of the machine learning model because they are commonly used evaluation metrics in the field. Accuracy of the whole model can also be calculated from values generated in a confusion matrix, and this provides some more information on the models. Figure 3 shows three confusion matrices with the corresponding TP, TN, FP, and FN values based on which class will be predicted using the machine learning model.

Precision refers to the percentage of instances the classifier labels as positive with respect to the total predictive positive instances, i.e., the TP divided by TP + FP (Equation (1)).

(1) $$\mathrm{Precision}=\frac{\mathrm{True}\;\mathrm{Positive}}{\mathrm{True}\;\mathrm{Positive}+\mathrm{False}\;\mathrm{Positive}}$$

A high precision score indicates that the model is making fewer false positive predictions. Precision is also known as positive predictive value (PPV). Recall refers to the proportion of events that actually were of a certain class that were classified as that class. It is derived by dividing the true positives to all positives, so it is derived by dividing the true positive by predicted results (Equation (2)).

(2) $$\mathrm{Recall}=\frac{\mathrm{True}\;\mathrm{Positive}}{\mathrm{True}\;\mathrm{Positive}+\mathrm{False}\;\mathrm{Positive}}$$

High recall score indicates that the model is identifying a larger number of actual positive examples. Recall is also known as sensitivity. The F1 score is a combination of precision and recall; it is the weighted average of the precision and recall scores (Equation (3)).

(3) $$F\text{-}1\;\mathrm{Score}=\frac{2}{\frac{1}{\mathrm{Precision}}+\frac{1}{\mathrm{Recall}}}=\frac{2\times \mathrm{Precision}\times \mathrm{Recall}}{\mathrm{Precision}+\mathrm{Recall}}$$

It ranges from 0 to 1. A value of 1 indicates perfect precision and recall, while a value of 0 indicates poor precision or recall. Overall accuracy refers to the sum of correctly classified values divided by the total number of values in the confusion matrix (Equation (4)).

(4) $$\mathrm{Accuracy}=\frac{\mathrm{True}\;\mathrm{Positive}+\mathrm{True}\;\mathrm{Negative}}{\mathrm{True}\;\mathrm{Positive}+\mathrm{False}\;\mathrm{Positive}+\mathrm{True}\;\mathrm{Negative}+\mathrm{False}\;\mathrm{Negative}}$$

## 3. Results

### 3.1. GTM Metrics

**Figure 4:**
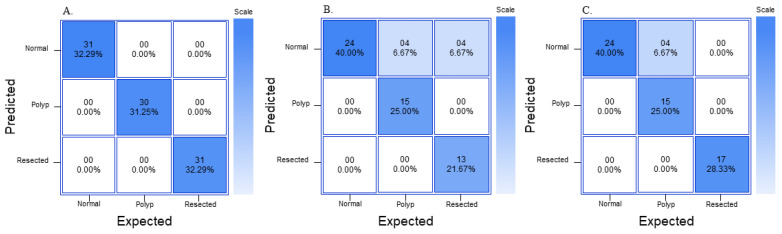
Normalized confusion matrix from (**A**) GTM, (**B**) RF3, and (**C**) YOLOv8n.

**Figure 5:**
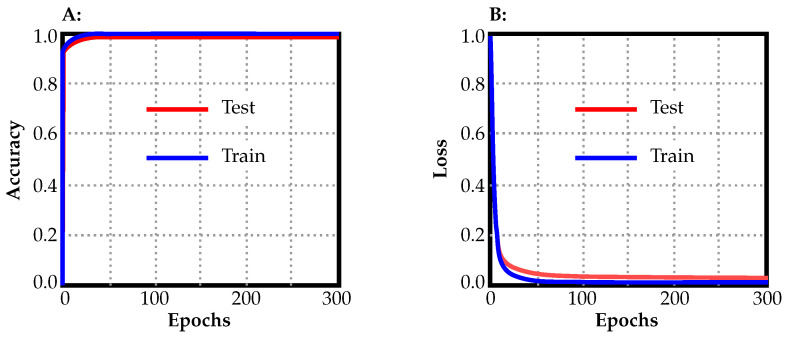
Accuracy (**A**) and loss (**B**) plots from GTM.

The GTM produced a confusion matrix, which was subsequently normalized to display values as percentages (Figure 4A). GTM does not generate other metrics, so the main metrics were generated from the confusion matrix, including precision, recall, and F1 score (Table 2). For the “normal” class, the precision was 1.00, recall was 1.00, and F1 score was 1.00. For the “polyp” class, the precision was 1.00, recall was 1.00, and F1 score was 1.00. For the “resected polyp” class, the precision was 1.00, recall was 1.00, and F1 score was 1.00. The overall accuracy of the GTM model was 1.00 (Table 3). Performance graphs generated by GTM showed that, as the number of epochs increased, the accuracy of the train and test split equally increased (Figure 5A). In addition, as the number of epochs increased, the loss of the train and test split decreased equally (Figure 5B). Loss represents how well the model is performing during training.

### 3.2. RF3 Metrics

**Figure 6:**
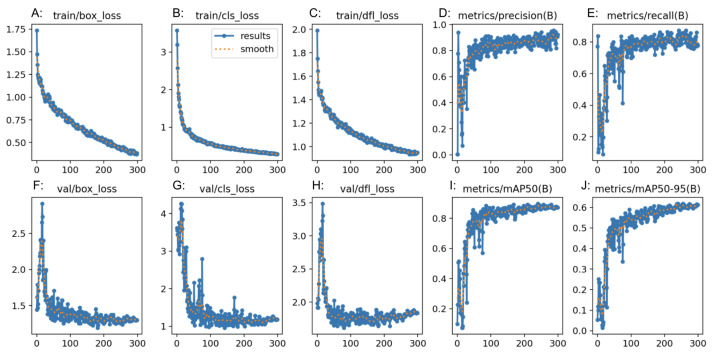
Precision (**D**,**I**,**J**), recall (**E**), and loss (**A**–**C**,**F**–**H**) plots from RF3.

The generated confusion matrix for RF3 was normalized to display values as percentages (Figure 4B). Further metrics, such as precision, recall, and F1 score, were generated from the confusion matrix (Table 4). For the “normal” class, the precision was 0.75, recall was 1.00, and F1 score was 0.86. For the “polyp” class, the precision was 1.00, recall was 0.79, and F1 score was 0.88. For the “resected polyp” class, the precision was 1.00, recall was 0.76, and F1 score was 0.87. Additional metrics from RoboFlow itself showed an average precision and mAP for all three classes as 0.89, with an average recall of 0.82. The overall accuracy of the RF3 model was 0.87 (Table 3). Performance graphs generated by RF3 showed that, as the number of epochs increased, the bounding box regression loss (box_loss) (Figure 6A,F), classification loss (cls_loss) (Figure 6B,G), and deformable convolution layer loss (dfl_loss) (Figure 6C,H) decreased. In addition, as the number of epochs increased, the precision, mAP (Figure 6D,I,J), and recall (Figure 6E) increased. “Box_loss” measures the error in predicting bounding box coordinates and dimension, so a lower “box_loss” indicates higher accuracy in detecting the location of polyps. “Cls_loss” refers to the error in the predicted class probabilities for each object in the image, so a lower “cls_loss” indicates higher accuracy of the model in predicting the class that an image belongs to (normal, polyp, or resected polyp in the current study). “Dfl_loss” measures the error in the deformable convolution layers, which allow the model to detect objects in images of different scales and aspect ratios, so a lower “dfl_loss” indicates that the model is better at handling images with different variations and aspect ratios.

### 3.3. YOLOv8n Metrics

**Figure 7:**
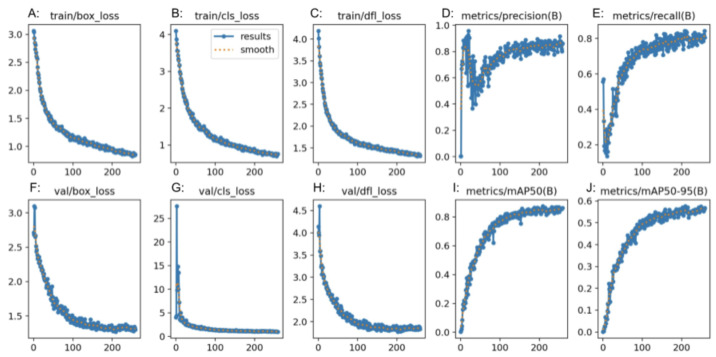
Precision (**D**,**I**,**J**), recall (**E**), and loss (**A**–**C**,**F**–**H**) plots from YOLOv8n.

The generated confusion matrix for YOLOv8n was normalized to display values as percentages (Figure 4C). Further metrics, such as precision, recall, and F1 score, were generated from the confusion matrix (Table 5). For the “normal” class, the precision was 0.86, recall was 1.00, and F1 score was 0.92. For the “polyp” class, the precision was 1.00, recall was 0.79, and F1 score was 0.88. For the “resected polyp” class, the precision was 1.00, recall was 1.00, and F1 score was 1.00. Additional metrics from YOLOv8n itself showed an average precision for all three classes of 0.90, mAP of 0.95, and an average recall of 0.90. The overall accuracy of the RF3 model was 0.84 (Table 3). The performance graphs generated by RF3 showed that, as the number of epochs increased, the bounding box regression loss (box_loss) (Figure 7A,F), classification loss (cls_loss) (Figure 7B,G), and deformable convolution layer loss (dfl_loss) (Figure 7C,H) decreased in both the training and validation plots. In addition, as the number of epochs increased, the precision, mAP (Figure 7D,I,J), and recall (Figure 7E) increased. The overall average accuracy, precision, recall, and F1 score among all three classes for GTM, RF3, and YOLOv8n are listed in Table 3.

### 3.4. External Validity Assessment

In assessing the external validity of the GTM model, the “normal”, “polyp”, and “resected polyp” classes had a precision, recall, and F1 score of 1.00 (Table 6). The average confidence in the prediction of the “normal” class in GTM was 99.70, in the “polyp” class was 98.13, and in the “resected polyp” class was 99.10. The overall accuracy of the GTM model was 1.00 (Table 7). The average confidence and standard deviation (SD) of prediction are also listed in Table 6. The average confidence was comparable among the different classes, but the “polyp” class had the highest SD.

For the RF3 model, the “normal” class had a precision of 0.91, recall of 1.00 and F1 score of 0.95 and a confidence of 93.26 (Table 8). The “polyp” class had a precision of 1.00, recall of 0.90, and F1 score of 0.95 and a confidence of 80.60. The “resected polyp” class had a precision of 1.00, recall of 1.00, and F1 score of 1.00 and a confidence of 89.97. The overall accuracy of the RF3 model is 0.97 (Table 7). The average confidence and standard deviation (SD) of prediction are also listed in Table 8. The average confidence was lower for the “polyp” class compared to the “normal” and “resected polyp” classes, with the “polyp” class having the highest SD compared to the other two classes.

**Figure 8:**
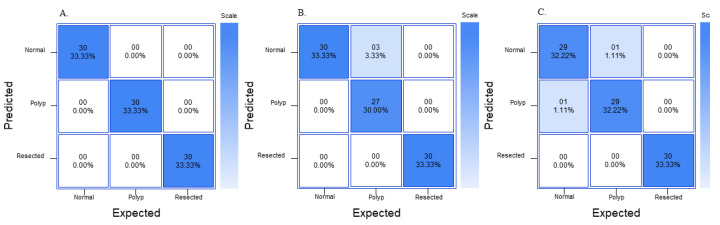
Normalized confusion matrix for external validity dataset with (**A**) GTM, (**B**) RF3, (**C**) YOLOv8n.

For the YOLOv8n model, the “normal” class had a precision of 0.97, recall of 0.97, and F1 score of 0.98 and a confidence of 85.5 (Table 9). The “polyp” class had a precision of 0.97, recall of 0.97, and F1 score of 0.98 and a confidence of 79.78. The “resected polyp” class had a precision of 1.00, recall of 1.00, and F1 score of 1.00 and a confidence of 75.73. The overall accuracy of the RF3 model is 0.97 (Table 7). The average confidence and SD of prediction are also listed in Table 9. The average confidence was lower for all three classes for the YOLOV8 model compared to the GTM and RF3 models, with the “polyp” and “resected polyp” having a lower average confidence than the “normal” class, and the “polyp” class had the highest SD compared to the other two classes. The confusion matrix from all three models on external validity testing is outlined in Figure 8.

## 4. Discussion

In the current study, the performance of three machine learning image classification models, GTM, RF3, and YOLOv8n, was compared in distinguishing between normal colon, polyps, and resected polyps in the colon. The aim of the study is to assess each model’s performance “out of the box”, without any user modifications. Metrics generated by GTM demonstrated excellent performance in classifying images into their respective classes. An analysis generated from the confusion matrix revealed the highest precision, recall, and F1 scores for all classes. The performance graphs generated by GTM indicated that, as the number of epochs increased during training, the accuracy and loss of both train and test splits improved simultaneously, suggesting that the model should theoretically be able to generalize its findings well on both the training and test data, and there are minimal signs of “overfitting” of the data. “Overfitting” in machine learning means that the model is memorizing features of images instead of trying to extrapolate the features that allow it to make generalizations on the image belonging to a particular class [25].

RF3 also demonstrated strong performance in the classification task but with slightly lower metrics compared to GTM. The confusion matrix analysis showed good precision and recall values for all classes. The F1 score was comparable for all three models and was satisfactory. The “normal” class had a lower precision than the rest of the classes, and the “polyp” and “resected polyp” classes had a lower recall than the “normal” class. This could be explained by the lower number of samples in the testing split for classes “polyp” (19 images) and “resected polyp” (17 images) as compared to the “normal” (24 images) class. The test split of images created by Roboflow did not evenly split the images between the three classes, so misclassifications can create bigger differences in the different metrics. Therefore, the metrics generated from the external validity test are more comparable between the three models. The plots generated by RF3 also show good performance with minimal signs of overfitting as the loss decreased equally for the training (Figure 6A–C) and validation (Figure 6F–H) plots as the number of epochs increased; however, the validation plots had some spikes in loss.

YOLOv8n also demonstrated competitive performance in the classification task, with metrics falling between those of GTM and RF3. The confusion matrix analysis showed good precision and recall values for all classes. The F1 score for all three classes was also better than the RF3 model but not as high as GTM. The “normal” class had a lower precision than the rest of the classes, and the “polyp” and “resected polyp” classes had a lower recall than the “normal” class. This is very similar to the trends found in the RF3 model, and this could be explained by the same reasoning of unevenly split images between the three classes. Therefore, the metrics generated from the external validity test are more comparable between the three models. The plots generated by YOLOv8n also show good performance, with lower signs of overfitting than the RF3 model as the loss decreased equally for the training (Figure 6A–C) and validation (Figure 6F–H) plots without any sudden spikes.

In the external validity assessment, all three models demonstrated robust generalization to new unseen data. GTM achieved perfect precision, recall, and F1 scores for all classes, along with a remarkable overall accuracy of 1.00. RF3 and YOLOv8n also performed well, with precision, recall, and F1 scores close to their original evaluation on the test set. Overall, the study revealed that GTM, RF3, and YOLOv8n are all capable of effectively classifying colon images into the categories of “normal”, “polyp”, and “resected polyp”. GTM exhibited outstanding performance, with high precision, recall, and F1 scores, as well as excellent external validity. RF3 and YOLOv8n also performed well, with competitive metrics and strong generalization to new data. The choice of the most suitable model may depend on specific use cases, computational resources, and the required level of precision and recall for the application. The metrics for both RF3 and YOLOv8n models (precision, recall, and F1 score) were comparable with each other; therefore, recommendations on model of choice are provided based on the usability and complexity of each model. For a beginner, the RF3 model is recommended as the interface is extremely user-friendly and easy to understand; however, all images will be published publicly if a user is not on the starter (USD 249/month) or enterprise plan (varies per team). If the end user uses patient images from a hospital database, it might violate the Health Insurance Portability and Accountability Act (HIPPA) in the United States if the images are made public without proper consent. Therefore, for any healthcare professional who would like to create a robust model on a custom dataset that they would not like to be published publicly, the YOLOv8n model is recommended. The YOLOv8n model also allows for a great deal of customization and is open-source; however, with this increased customizability also comes increased complexity. GTM, even though it had perfect metrics, would not be recommended for detection as it can only classify images but does not detect where the polyp is in an image. In addition, although GTM may not exhibit signs of overfitting, it does not guarantee that it can effectively generalize to images from other databases with different lighting and quality.

Comparing all three models, GTM demonstrated exceptional performance in the classification task, achieving perfect precision, recall, and F1 scores for all classes on both the original test set and the external validation set. GTM’s key advantages include its ease of use and accessibility, making it an excellent option for researchers or practitioners without extensive machine learning expertise. GTM is a classification model, not a detection model; therefore, its architacture is simpler than the RF3 and YOLOv8n models, which are detection models. The high accuracy, precision, recall, and F1-score of the GTM model could be explained by the fact that it classifies the different images into “normal”, “polyp”, or “resected”. However, RF3 and YOLOv8n detect the location of the polyp in the image itself. In addition, all of the “resected polyp” images from the HyperKvasir dataset [22] were dyed blue; therefore, the GTM model could theoretically use the color data to classify the resected polyps correctly. GTM was still able to correctly classify between the “normal” and “polyp” classes even though they had similar-looking backgrounds. Many gastroenterologists dye polyps blue before polyp resection for better visualization; therefore, this is clinically relevant. RF3 performed well, with competitive metrics on both the original test set and the external validation set. One of the significant advantages of RF3 is its ability to handle more complex tasks thanks to its capability for bounding box regression and object detection using deformable convolution layers. YOLOv8n also displayed competitive performance, with metrics falling between those of GTM and RF3. The better performance shown by the YOLOv8n model as compared to the RF3 model could be indicated by the fact that this was the only model with augmentation steps added, thus possibly biasing the results for this model.

Despite their strengths, all three models exhibited certain limitations that merit consideration. GTM’s main limitation is its relatively simple architecture, which might not be suitable for more complex image classification tasks. In addition, GTM is a classification model, whereas the RF3 and YOLOv8n models used are image detection programs that detect the polyp rather than simple classification. Image classification refers to the process of categorizing images into specific classes or categories based on their visual content. This task is essential for applications such as object recognition, scene understanding, and image retrieval. On the other hand, image detection focuses on localizing and identifying specific objects or regions within an image [26]. Compared to image classification, which only requires determining the category or class of an entire image, image detection involves identifying and localizing objects within an image.

YOLOv8n relies on more complex architectures that demand substantial computational resources and longer training times [27]. Additionally, these models may require more fine-tuning and parameter adjustments to achieve optimal performance, making them less user-friendly for those without extensive machine learning expertise. However, the outcomes of RF3 and YOLOv8n are more applicable for clinical practice as they can be used to ensure that colonic polyps are not missed. Comparing the RF3 and YOLOv8n models, the YOLOv8n model can be used remotely or in a private Google Drive. For RF3, for a free user, all the images that will be used for training, validation, and testing must be uploaded onto the RoboFlow website, and they are not private. In addition, free users also only receive a limited amount of credits to generate RF3 models. However, RoboFlow is extremely user-friendly and requires no understanding of coding to implement via the RoboFlow website, whereas YOLOv8n requires some understanding of coding. During the current study, it took approximately 25 min to train the model using RF3, 51 min to train the YOLOv8n model, and only 2 min to train the GTM model. GTM was the fastest in training, followed by RF3 and YOLOv8n as YOLOv8n took almost double the time as RF3. However, this could be explained by differences in the hardware used as the RF3 model was developed and trained on Roboflow-hosted GPUs online and the YOLOv8n model was trained on the free version of Google Colaboratory with Tesla T4 GPU. Comparing the different models provides more information on how the different models operate and how each one can be fine-tuned or customized to improve the metrics. Therefore, future research could focus on optimizing and fine-tuning the hyperparameters of RF3 and YOLOv8n to enhance their performance while considering the computational cost. Furthermore, exploring ensemble methods that combine the strengths of different models could potentially lead to even better results. In addition, cross-validation techniques such as the k-fold approach can be used to evaluate a model’s ability to generalize to new data and mitigate overfitting. Finally, further studies can also be conducted on how each model reacts to multiple instances of testing for each class.

Previous studies by Chen et al. on detecting polyps using the DeFrame system have shown 100% recall and 80% sensitivity [13]. The machine learning polyp detection model built on the YOLOv5 architecture created by Wan et al. achieved a recall of 90% and precision of 91% [16]. In addition, a study conducted by Tanwar et al. on detecting polyps using a Single Shot MultiBox Detector had a sensitivity of 96% and accuracy of 86% [19]. Compared to the aforementioned models, the best detection model in the current study (YOLOv8n) had an average accuracy of 87%, precision of 95%, and recall of 93%. However, the current study was conducted on a smaller subset of the HyperKvasir and thus is not a one-to-one comparison to the above studies. In addition, the above studies use different datasets with different images, thus providing other variables that are not controlled.

Machine learning image detection systems hold profound clinical significance in the realm of colonic polyp detection. Adenomatous polyps serve as potential precursors to colorectal cancer, making their early detection and removal essential for the prevention of progression to colorectal cancer. Colonoscopy is the gold standard screening test to detect early colorectal cancer and colonic polyps. Limitations in colonoscopy procedures include a high polyp miss rate for small (<10 mm) or flat polyps, which can be easily missed during visual inspection [28]. The miss rate for colonic polyps varies from 6 to 27% [29]. Colonoscopies used to be heavily endoscopic-dependent until the use of computer-aided detection systems developed in 1966. Computer-aided detection systems have made a huge impact in aiding endoscopists to detect suspicious polyps. Artificial-intelligence-assisted systems can be a crucial tool in detecting polyps due to some challenges in traditional screening methods, such as suboptimal procedure techniques and the subjectivity of the endoscopist. Artificial-intelligence-assisted systems are still in an area of rapid change. This current study is a proof of concept for a machine learning model that can detect colonic polyps. Future studies will improve upon the current models and compare it to state-of-the-art polyp detection models that are used in a clinical setting. The current study aims to help move this research in polyp detection in colonoscopies in order to figure out the best machine learning model to use for detection of colonic polyps.

## 5. Conclusions

In conclusion, the comparison of GTM, RF3, and YOLOv8n revealed that each model possesses unique advantages and limitations. GTM is exceptionally user-friendly and effective for quick prototyping, while RF3 excels in more complex tasks with precise object detection. YOLOv8n offers computational efficiency and real-time capabilities, making it suitable for time-sensitive applications. The choice of the most appropriate model will depend on the specific requirements of the image classification task, available resources, and the desired level of accuracy and efficiency.

Some of the limitations for the current study included the relatively small sample size, potentially affecting the generalizability of the results. In addition, the images for this study were sourced from one dataset and this could impact the models’ real-world applicability. In addition, the “normal” colonic tissue images for the current study were sourced from the cecum of the colon as the “normal” mucosa images from the “HyperKvasir” dataset came from the cecum of the colon.

Future direction for the current study involves increasing the sample size and utilizing a wide variety of images from different resources for better generalizability. The current study can also be made more clinically relevant by using dyed polyp images versus dyed resected polyp images in order to create a model that can detect whether a polyp has been completely removed during colonoscopy. Additionally, external data augmentation strategies will also be employed to increase the dataset’s size and diversity. Finally, an ablation process can also be implanted to systematically analyze the impact of different components of the model on the overall performance of the model, thus shedding light on the key factors influencing the metrics of the model.

While the performance metrics for all models appear favorable, it is crucial to consider the broader context of usability, complexity, and suitability for various applications. GTM offers an accessible entry point for beginners but is a simple classification model over a detection model. RF3 provides a good combination of performance and user-friendliness. However, it should be noted that the free version of Roboflow does not offer privacy protection for data, which may make it unsuitable for handling private patient information. YOLOv8n excels in real-time applications but demands a deeper understanding of coding. The recommendations for model selection are rooted in the specific needs of users, from healthcare professionals to researchers, and consider different factors, such as privacy regulations and computational resources.

## Figures and Tables

**Table 1:** Summary of epochs, batch size, and learning rate for all models.

Model	Epochs	Batch Size	Learning Rate
GTM	300	32	0.0001
YOLOv8n	300	32	0.001429
RF3	300	–	–

**Table 2:** Precision, recall, and F1 score for GTM.

Class	Precision	Recall	F1 Score
Normal	1.00	1.00	1.00
Polyp	1.00	1.00	1.00
Resected Polyp	1.00	1.00	1.00

**Table 3:** Average accuracy, precision, recall, and F1 score between all three classes for GTM, YOLOv8n, and RF3.

Model	Accuracy	Precision	Recall	F1 Score
GTM	0.99	1.00	1.00	1.00
YOLOv8n	0.87	0.95	0.93	0.93
RF3	0.84	0.92	0.85	0.87

**Table 4:** Precision, recall, and F1 score for RF3.

Class	Precision	Recall	F1 Score
Normal	0.75	1.00	0.86
Polyp	1.00	0.79	0.88
Resected Polyp	1.00	0.76	0.87

**Table 5:** Precision, recall, and F1 score for YOLOv8n.

Class	Precision	Recall	F1 Score
Normal	0.86	1.00	0.92
Polyp	1.00	0.79	0.88
Resected Polyp	1.00	1.00	1.00

**Table 6:** Precision, recall, and F1 score for GTM for external validation dataset.

Class	Precision	Recall	F1 Score	Average Confidence ± SD
Normal	1.00	1.00	1.00	99.70 ± 1.32
Polyp	1.00	1.00	1.00	98.13 ± 7.65
Resected Polyp	1.00	1.00	1.00	99.10 ± 4.24

**Table 7:** Accuracy measurement differences from GTM, RF3, and YOLOv8n for external validation dataset.

Model	Accuracy
GTM	1.00
RF3	0.97
YOLOv8n	0.97

**Table 8:** Precision, recall, and F1 score for RF3 for external validation dataset.

Class	Precision	Recall	F1 Score	Average Confidence ± SD
Normal	0.91	1.00	0.95	93.26 ± 1.68
Polyp	1.00	0.90	0.95	80.60 ± 28.81
Resected Polyp	1.00	1.00	1.00	89.97 ± 5.00

**Table 9:** Precision, recall, and F1 score for YOLOv8n for external validation dataset.

Class	Precision	Recall	F1 Score	Average Confidence ± SD
Normal	0.97	0.97	0.98	85.50 ± 16.80
Polyp	0.97	0.97	0.98	79.78 ± 20.29
Resected Polyp	1.00	1.00	1.00	75.73 ± 11.73

## Data Availability

Data is contained within the article. Name: HyperKvasir, Dataset Link: https://doi.org/10.6084/m9.figshare.12759833 accessed on 28 September 2023, Affiliation: Scientific Data Curation Team.

## Abbreviations

The following abbreviations are used in this manuscript:

GI	Gastrointestinal
GTM	Google Teachable Machine
RF3	Roboflow 3.0 Object Detection
YOLOv8n	You Only Look Once version 8
CVAT	Computer Vision Annotation Tool
MS COCO v7	Microsoft Common Objects in Context version 7
Colab	Google Colaboratory
YAML	YAML Ain’t Markup Language
CLAHE	Contrast Limited Adaptive Histogram Equalization
AI	Artificial Intelligence
JPEG	Joint Photographic Experts Group
YOLOv8nn	YOLOv8n nano
AdamW	Adaptive Moment Estimation version W optimizer
PPV	Positive Predictive Value
mAP	Mean Average Precision
box_loss	Bounding Box Regression Loss
cls_loss	Classification Loss
dfl_loss	Deformable Convolution Layer Loss
CNNs	Convolutional Neural Networks
CLAHE	Contrast Limited Adaptive Histogram Equalization
TP	True Positive
TN	True Negative
FP	False Positive
FN	False Negative
SD	Standard Deviation
HIPPA	Health Insurance Portability and Accountability Act
